# Problematic internet use among young people in Türkiye: a meta-analysis

**DOI:** 10.3389/fpsyg.2026.1885952

**Published:** 2026-10-30

**Authors:** Gökhan Kayır, Bekir Güzel

**Affiliations:** 1 Faculty of Education, Manisa Celal Bayar University, Manisa, Türkiye; 2 Stiftung Zentrum für Türkeistudien und Integrationsforschung (ZfTI), University of Duisburg-Essen, Essen, Germany; 3 Department of Social Work, Faculty of Health Science, Recep Tayyip Erdoğan University, Rize, Türkiye

**Keywords:** adolescents, emerging adults, meta-analysis, problematic internet use, public health, Türkiye

## Abstract

**Background:**

Problematic internet use (PIU) is a growing public health concern among young people, yet meta-analytic evidence from Türkiye has largely focused on gender differences. This meta-analysis examined gender differences in PIU among young people in Türkiye, as well as associations with daily internet use and family income.

**Methods:**

Following the PRISMA 2020 guidelines, Researchers searched Web of Science, Scopus, TR Dizin, and Google Scholar, including the cited-by lists of the scale development article, for quantitative studies that measured PIU with the Problematic Internet Use Scale or its adolescent form in high school or university students. Research included 27 studies (17 graduate theses and 10 journal articles) published between 2010 and 2025 (*N* = 16,488). Researchers computed effect sizes as Hedges’ g and pooled them using random-effects models with restricted maximum likelihood estimation and the Knapp–Hartung adjustment. Publication year and educational level were tested as moderators, and small-study effects were examined with funnel plots, Egger’s regression test, and the trim-and-fill method.

**Results:**

Male students reported higher PIU than female students (*k* = 27; *g* = 0.28, 95% CI [0.18, 0.38]), and this difference was smaller in more recent studies. Students in the longest daily-use category reported substantially higher PIU than those in the shortest category (*k* = 10; *g* = 1.31, 95% CI [1.03, 1.60]), and the association remained large after trim-and-fill adjustment. The difference between the highest and the lowest family-income categories was small and not statistically significant (*k* = 8; *g* = 0.16, 95% CI [−0.08, 0.39]). Heterogeneity was high for gender and daily use and moderate for family income.

**Conclusion:**

Among young people in Türkiye, PIU is most strongly associated with time spent online; the gender difference is small and appears to be narrowing, and the evidence regarding family income is inconclusive. Because all included studies were cross-sectional, the findings describe associations rather than causal effects. Prevention programs may benefit from addressing daily patterns of internet use, and future studies should test theory-based mechanisms with longitudinal designs.

## 1 Introduction

Rapid developments in information and communication technologies have transformed everyday life, and young people are among the most intensive internet users. According to the Turkish Statistical Institute (TURKSTAT, 2025), the proportion of internet users among individuals aged 16–24 in Türkiye reached 96.5% in 2024 (97.4% for men and 95.6% for women), compared with 26.6% in 2004 (38.3% for men and 15.9% for women). A similar trend is observed elsewhere: in the European Union, 97% of young people used the internet in 2023, compared with 86% of the general population (EUROSTAT, 2025). Alongside these benefits, growing internet use has drawn attention to problematic internet use (PIU).

PIU refers to a loss of control over internet use that disrupts daily activities and is accompanied by psychological and social difficulties (Ceyhan et al., 2007). Adolescents and young adults are considered particularly vulnerable because of their developmental characteristics (Özgözgü et al., 2022). Studies have identified several factors associated with PIU, including easy access to the internet, limited social support, and family structure (Alevsaçanlar Cücü, 2020). PIU has also been associated with lower academic achievement, social isolation, and psychological problems (Aras and Karakuş, 2025; Günlü and Ceyhan, 2017), with time loss, exposure to misinformation, and difficulties in self-control (Turanalp, 2016), with emotional loneliness and low self-esteem (Yaşar, 2021), and with anxiety, stress, and depressive symptoms (Aras and Karakuş, 2025; Dinç, 2015).

Daily internet use among young people in Türkiye has been reported to average about 7.5 h, much of it devoted to social media, online games, and other digital content (Aras and Karakuş, 2025); comparable figures are about 6 h and 40 min worldwide (We Are Social, 2025) and more than 7 h in the United States and the United Kingdom (Backlinko, 2025). Higher PIU has been associated with musculoskeletal complaints, lower academic performance, poor time management, depression, anxiety, and stress among adolescents (İkiz et al., 2015; Moreno et al., 2011; Vlad, 2024), and intensive internet use has been linked to risks such as cyberbullying, illegal betting and fraud (Gönültaş, 2022; Kusumadewi et al., 2025). For these reasons, PIU is increasingly regarded as a public health concern (Chen et al., 2024; Theodoratou et al., 2025; Yildirim Demirdöğen et al., 2024).

Adolescence and emerging adulthood, the latter spanning roughly the late teens to the mid-twenties (Arnett, 2000), are periods when identity exploration, peer orientation, and growing autonomy coincide with intensive digital media use. The 15–24 age band examined in this study covers late adolescence and the first years of emerging adulthood. Although these periods differ developmentally, in the Turkish education system they largely correspond to high school and undergraduate students, who share comparable conditions of internet access and use; therefore, researchers examined educational level as a moderator rather than assuming it was irrelevant.

Theoretical accounts help explain why demographic and behavioral characteristics may relate to PIU. Davis's (2001) cognitive-behavioral model distinguishes specific from generalized pathological internet use and proposes that distal vulnerabilities set the stage for PIU. In contrast, maladaptive cognitions about the self and the online world, such as the belief that one is valued only online, and the reinforcement obtained online maintain it. The Interaction of Person-Affect-Cognition-Execution (I-PACE) model extends this view: it describes problematic use as the product of interactions between predisposing person characteristics, affective and cognitive responses to triggers, executive control, and the gratification or relief obtained through use, which become stronger as use is repeated (Brand et al., 2016; Brand et al., 2019). Within this framework, gender and family socioeconomic conditions can be viewed as distal person and environmental characteristics that shape usage motives, access, and parental mediation. In contrast, daily time online reflects the behavioral pattern through which reinforcement accumulates. A recent narrative theoretical review of research on adolescents and emerging adults likewise emphasized that associations between digital media use and mental health, including problematic use, vary with support factors, risk behaviors, and moderating variables (Luis et al., 2026).

Research on PIU in Türkiye has generally examined a limited number of demographic correlates. Most studies report higher PIU among male than female students, although findings are mixed and some studies report no significant gender difference; younger students and students in lower academic years have also been found to report higher PIU (Akgün Kostak et al., 2019; Aktu, 2024). Higher household income, ownership of internet-enabled devices, and private internet access have been associated with a higher risk of PIU (Sayılı et al., 2021). Previous meta-analyses conducted in Türkiye focused exclusively on gender (Akbaş et al., 2019; Öksüz, 2019). To our knowledge, no meta-analysis has synthesized differences in PIU by daily internet use or family income, or examined whether the gender difference has changed over time.

The present study therefore aimed to synthesize differences in PIU among young people in Türkiye by gender, daily internet use, and family income, using studies that measured PIU with the Problematic Internet Use Scale (Ceyhan et al., 2007) or its adolescent form (Ceyhan and Ceyhan, 2014). The following research questions guided the study:

1. Do PIU scores differ between male and female young people?
2. Do PIU scores differ between young people in the longest and the shortest daily internet use categories?
3. Do PIU scores differ between young people from families in the highest and the lowest income categories?

We also examined whether publication year and educational level (high school vs. university) moderate these differences.

## 2 Methods

### 2.1 Reporting standards and protocol

This systematic review and meta-analysis was conducted and reported in accordance with the PRISMA 2020 statement (Page et al., 2021); the completed checklist is provided as Supplementary file S2. The review protocol was not registered in advance, which is acknowledged as a limitation.

### 2.2 Eligibility criteria

Eligibility criteria were specified with the PECOS framework, an adaptation of PICO for observational evidence (Morgan et al., 2018):

*Population*: young people in Türkiye enrolled in high school (Grades 9–12) or undergraduate programs, broadly corresponding to the 15–24 age band commonly used to define youth in international statistics. Samples drawn from middle schools were excluded.

*Exposure/comparison*: gender (male vs. female), daily internet use (longest vs. shortest reported duration category), and family income (highest vs. lowest reported income category).

*Outcome*: PIU measured with the Problematic Internet Use Scale (PIUS; Ceyhan et al., 2007) or its adolescent form (PIUS-A; Ceyhan and Ceyhan, 2014). Use of the scale was verified in the full text; studies that cited the scale but measured PIU with another instrument were excluded.

*Study design*: quantitative cross-sectional (correlational or causal-comparative) studies reporting group sizes, means, and standard deviations for at least one comparison, irrespective of the significance test applied.

Peer-reviewed journal articles and master’s or doctoral theses written in Turkish or English were eligible. When the same data set appeared in more than one record, the most completely reported record was retained. Studies were required to report reliability evidence of at least 0.70 for the PIUS, either a sample-specific Cronbach’s alpha or a coefficient from the scale’s validation studies; studies reporting no reliability information were excluded. Whether a sample-specific coefficient was reported was coded as a quality indicator (Section 2.6).

### 2.3 Information sources and search strategy

Records were identified through searches of Web of Science, Scopus, TR Dizin, and Google Scholar, combined with the “cited by” lists of the scale development article (Ceyhan et al., 2007) in these sources; the full texts of graduate theses were retrieved from the National Thesis Center of the Council of Higher Education (YÖK). Because citation lists also include studies that cite the scale without administering it, we verified the instrument used in each study in the full text. Because Google Scholar also indexes non-peer-reviewed outlets, the indexing status of every included journal article was checked and is reported in Supplementary Table S1; all included theses are approved graduate theses archived by YÖK.

### 2.4 Study selection and data extraction

Titles and abstracts were screened first, and potentially eligible records were assessed in full text against the criteria above. During revision, every included record was re-examined against its full text, and all effect-size data were re-extracted from the primary source tables. Discrepancies between the initial coding and the primary sources, such as transposed group means, omitted response categories, or misattributed references, were corrected; each correction is documented in Supplementary Table S1.

The following information was extracted from each study: author(s) and year, publication type, city and institution, educational level, age range, sample size by gender, scale form (PIUS or PIUS-A), sample-specific reliability, the response categories reported for daily internet use and family income, and the group sizes, means and standard deviations required for each comparison.

### 2.5 Effect size computation

Standardized mean differences were computed as Hedges’ *g*, which applies a small-sample correction to Cohen’s *d* (Borenstein et al., 2009). Positive values indicate higher PIU scores among male students, among students in the longest daily-use category, and among students in the highest family-income category. Because the PIUS (33 items, range 33–165) and the PIUS-A (27 items, range 27–135) differ in score range, and one study reported scores transformed to a 20–100 metric, standardized effect sizes were required to place all studies on a common scale.

The included studies used different response categories for daily internet use and family income (e.g., “less than 1 hour” to “5 hours or more”; income brackets in Turkish lira that changed with inflation across years). To apply a single rule to all studies, the highest and the lowest reported categories were compared within each study. Categories such as “never” or “do not know” were not treated as duration categories. Two studies reported weekly rather than daily hours of use; they were retained and examined in a sensitivity analysis. Each study contributed one effect size per analysis.

### 2.6 Methodological quality indicators

No formal risk-of-bias instrument was applied. Instead, three indicators relevant to the validity of the pooled estimates were coded: whether a sample-specific reliability coefficient for the PIUS was reported (12 of 27 studies; the remaining 15 reported coefficients from the validation studies only), publication type (journal article or thesis), and whether all extracted values could be verified against the full text (25 of 27 studies; for two studies only the abstract or extended summary was available). These indicators were used to define the sensitivity analyses described below.

### 2.7 Statistical analysis

Effect sizes were pooled with random-effects models estimated by restricted maximum likelihood (Viechtbauer, 2005), and confidence intervals and tests used the Knapp–Hartung adjustment (IntHout et al., 2014; Knapp and Hartung, 2003). Heterogeneity was assessed with Cochran’s *Q*, *τ*^2^, and *I*^2^, interpreted with the conventional benchmarks of 25%, 50%, and 75% (Higgins et al., 2003), and 95% prediction intervals were reported to describe the range of true effects expected in comparable settings (Riley et al., 2011).

Publication year and educational level (high school vs. university) were examined as moderators in separate mixed-effects meta-regressions. Robustness was assessed with leave-one-out analyses and with sensitivity analyses restricted to (a) studies whose values were fully verified against the primary source, (b) studies reporting a sample-specific reliability coefficient, (c) studies reporting daily rather than weekly hours (daily-use analysis), and (d) the originally coded set of studies (gender analysis).

Small-study effects were examined with funnel plots, Egger’s regression test, implemented as a meta-regression on the standard error (Egger et al., 1997), and the trim-and-fill method (Duval and Tweedie, 2000). Because these procedures have limited power when fewer than ten studies are available, their results for the family-income analysis were interpreted with caution (Sterne et al., 2011).

All analyses were conducted in R version 4.3.3 (R Core Team, 2024) using the metaphor package version 4.4–0 (Viechtbauer, 2010), with two-tailed tests at *α* = 0.05. The coded dataset and analysis script are provided as Supplementary files S3, S4.

## 3 Results

### 3.1 Study selection

The searches identified 312 records (Figure 1). Before screening, 25 duplicates, 13 records flagged as ineligible by automation tools, and 11 records removed for other reasons were excluded, leaving 263 records for title and abstract screening. Of these, 185 were excluded because they did not examine gender, daily internet use or family income, and 78 reports were assessed in full text. At the initial full-text assessment, 32 reports were excluded because they did not report the group sizes, means and standard deviations required to compute effect sizes, and 11 because they reported no reliability information for the PIUS. In the re-assessment conducted during revision, eight further reports were excluded. Five measured PIU with instruments other than the PIUS (Akgün Kostak et al., 2019; Durmuş et al., 2018; Esen, 2010; Odacı and Kalkan, 2010; Tutgun et al., 2011), one sampled middle-school students aged 12–14 years (Karasu et al., 2023), one reported no gender comparison of PIU scores (Eroğlu et al., 2023), and one entry duplicated the data of an included thesis (Duman, 2016). Twenty-seven studies were included in the meta-analysis.

**Figure 1:**
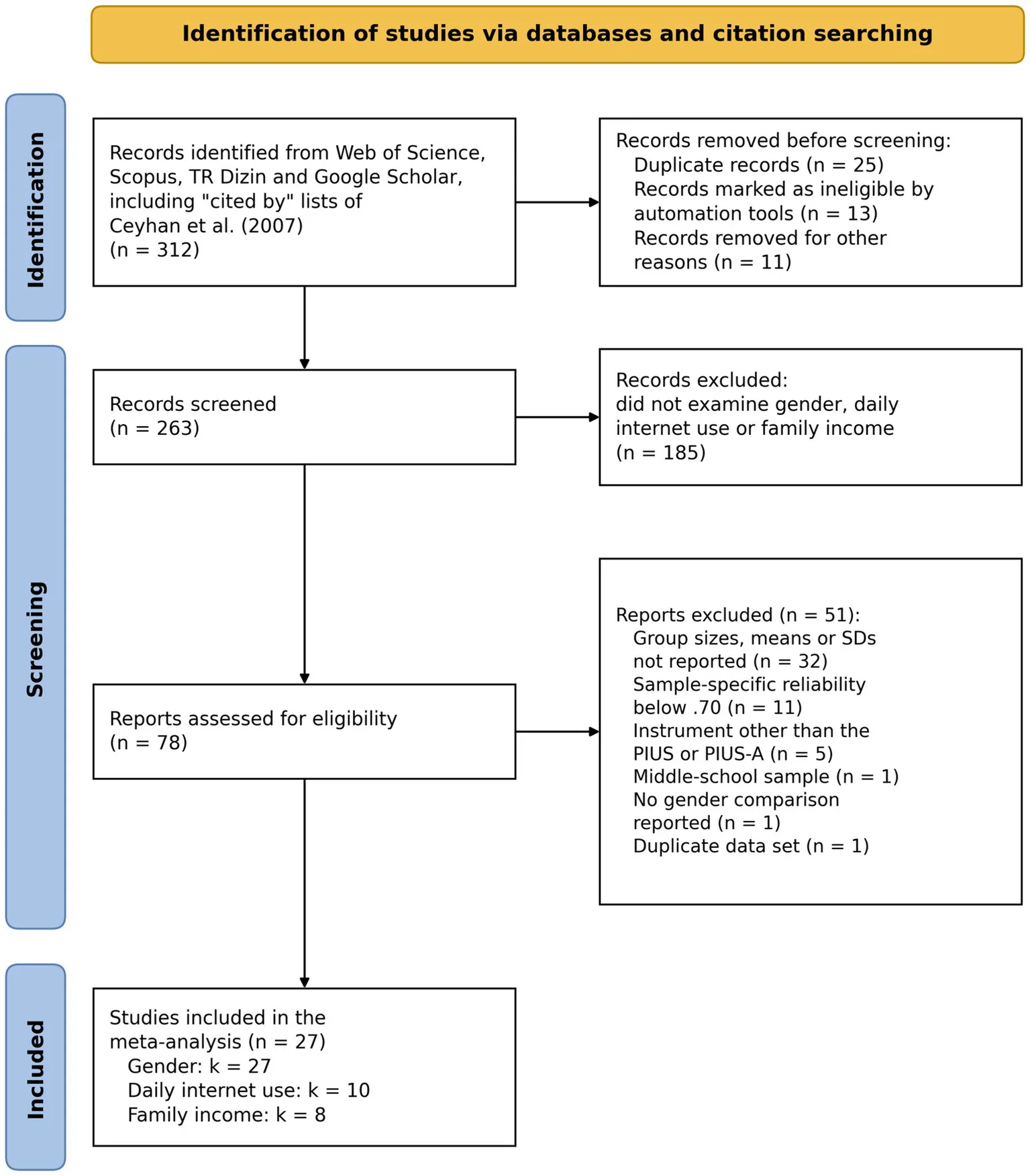
PRISMA 2020 flow diagram of study identification, screening, and inclusion.

### 3.2 Characteristics of the included studies

The 27 studies were published between 2010 and 2025 and comprised 16,488 participants (Table 1). Seventeen were graduate theses (16 master’s and one doctoral), and 10 were journal articles. Twenty studies sampled high school students and seven sampled university students; 13 used the PIUS and 13 the PIUS-A, and in one thesis the form could not be determined. Nine studies were conducted in İstanbul and 15 in 12 other provinces; for three studies, the province was not stated (Meral and Bahar, 2016; Saruhan and Çiftçi, 2025; Sırakaya and Seferoğlu, 2013). Twelve studies reported a sample-specific reliability coefficient for the PIUS, ranging from 0.86 to 0.96, and the remaining 15 reported coefficients from the validation studies. All 27 studies contributed to the gender analysis, 10 to the daily-use analysis, and eight to the family-income analysis.

**Table 1:** Characteristics of the included studies (*k* = 27).

Study	Type	Province	Level	N (F/M)	Scale	*α*	*g* gender [95% CI]	*g* daily use [95% CI]	*g* income [95% CI]
Ağca (2021)	MA thesis	İstanbul	High school	459 (330/129)	PIUS-A	—	0.06 [−0.14, 0.26]	—	0.31 [−0.14, 0.77]
Alçay (2019)	MA thesis	Antalya	High school	485 (247/238)	PIUS-A	0.93	0.32 [0.14, 0.49]	—	—
Altunten (2019)	MA thesis	İzmir	High school	365 (187/178)	PIUS-A	0.86	0.10 [−0.11, 0.30]	—	0.06 [−0.44, 0.55]
Arslankara and Usta (2020)	Article	Ağrı	High school	390 (176/214)	PIUS	—	0.46 [0.26, 0.66]	1.70 [1.19, 2.22]	—
Baylyyeva (2019)	MA thesis	İstanbul	High school	1,459 (1,084/375)	PIUS	—	−0.17 [−0.28, −0.05]	—	−0.29 [−0.79, 0.21]
Berber Çelik et al. (2015)	Article	Trabzon	University	314 (147/167)	PIUS	—	0.04 [−0.18, 0.26]	—	—
Büyükfiliz (2016)	MA thesis	İstanbul	University	251 (153/98)	PIUS	—	1.05 [0.79, 1.32]	1.44 [1.01, 1.87]^w^	0.46 [0.16, 0.76]
Çınar (2015)	MA thesis	İstanbul	High school	294 (123/171)	PIUS	—	0.31 [0.07, 0.54]	0.76 [0.40, 1.12]	—
Duman (2016)	MA thesis	Konya	High school	392 (242/150)	PIUS-A	—	0.41 [0.20, 0.61]	—	—
Durak et al. (2018)†	Article	İstanbul	High school	410 (165/245)	PIUS-A	—	−0.23 [−0.43, −0.03]	—	—
Durmuş and Başarmak (2014)	Article	Kırşehir	University	343 (219/124)	PIUS	—	0.47 [0.24, 0.69]	1.47 [1.07, 1.86]	—
Gürcan (2010)	MA thesis	Konya	High school	710 (330/380)	PIUS	—	0.44 [0.29, 0.59]	—	—
Kapudere (2020)	MA thesis	Gaziantep	University	419 (258/161)	PIUS	0.92	0.21 [0.02, 0.41]	0.71 [0.51, 0.91]	—
Meral and Bahar (2016)^†^	Article	NR	High school	242 (133/109)	PIUS-A	0.92	0.48 [0.22, 0.73]	—	—
Odacı and Berber Çelik (2012)	Article	Trabzon	University	661 (326/335)	PIUS	—	0.38 [0.23, 0.53]	—	—
Özer (2013)	MA thesis	Trabzon	High school	385 (203/182)	PIUS-A	0.93	0.52 [0.32, 0.72]	—	—
Öztabak (2023)	Article	İstanbul	High school	317 (189/128)	PIUS	0.95	0.05 [−0.18, 0.27]	—	—
Saruhan and Çiftçi (2025)	Article	NR	High school	1,596 (833/763)	PIUS-A	0.92	0.20 [0.10, 0.30]	—	—
Sevindik (2011)	PhD thesis	Elazığ	University	1,723 (563/1160)	PIUS	—	0.45 [0.35, 0.55]	—	0.44 [0.27, 0.62]
Sırakaya and Seferoğlu (2013)	Article	NR	University	226 (125/101)	PIUS	—	0.37 [0.11, 0.64]	1.45 [0.91, 1.99]	—
Şahan and Eraslan Çapan (2017)	Article	Eskişehir	High school	312 (174/138)	PIUS-A	0.96	0.37 [0.14, 0.59]	—	—
Şenyiğit (2018)	MA thesis	Mersin	High school	1,642 (812/830)	PIUS-A	0.89	0.03 [−0.06, 0.13]	—	—
Türkoğlu (2013)	MA thesis	İstanbul	High school	540 (307/233)	PIUS	—	0.55 [0.37, 0.72]	1.66 [1.24, 2.07]	−0.30 [−0.84, 0.24]
Yavuzarslan Gök (2017)	MA thesis	İstanbul	High school	215 (144/71)	PIUS-A	—	0.07 [−0.21, 0.35]	1.37 [0.97, 1.76]^w^	—
Yıldız (2010)	MA thesis	Sivas	High school	1,000 (512/488)	Unclear^‡^	0.93	0.38 [0.26, 0.51]	1.84 [1.52, 2.16]	0.08 [−0.18, 0.33]
Yüce (2018)	MA thesis	İstanbul	High school	656 (386/262)^a^	PIUS-A	0.90	−0.02 [−0.18, 0.13]	1.00 [0.79, 1.20]	—
Zorbaz (2013)	MA thesis	Ankara	High school	682 (356/326)	PIUS-A	0.92	0.37 [0.22, 0.52]	—	0.05 [−0.29, 0.39]

Note. All studies used cross-sectional survey designs. N = analyzed sample (female/male). *α* = sample-specific Cronbach’s alpha for the PIUS; — = not reported or not applicable. Hedges’ *g* > 0 indicates higher PIU among male students, among students in the longest daily-use category, and among students in the highest family-income category; daily use and income compare the extreme categories reported in each study. NR = province not stated. ^†^Values could not be fully verified against the full text (abstract or extended summary only). ^‡^The thesis describes both the 33-item PIUS and the PIUS-A.

w Weekly rather than daily hours of internet use.

a Gender data were reported for 648 participants.

### 3.3 Gender

Male students reported higher PIU than female students, with a small pooled effect (*k* = 27, *N* = 16,479; *g* = 0.28, 95% CI [0.18, 0.38], *p* < 0.001; Figure 2, Table 2). Heterogeneity was high (*Q*(26) = 215.07, *p* < 0.001; *I*^2^ = 88.8%; *τ*^2^ = 0.056), and the 95% prediction interval ranged from −0.22 to 0.78, indicating that the direction of the difference may vary across settings.

**Figure 2:**
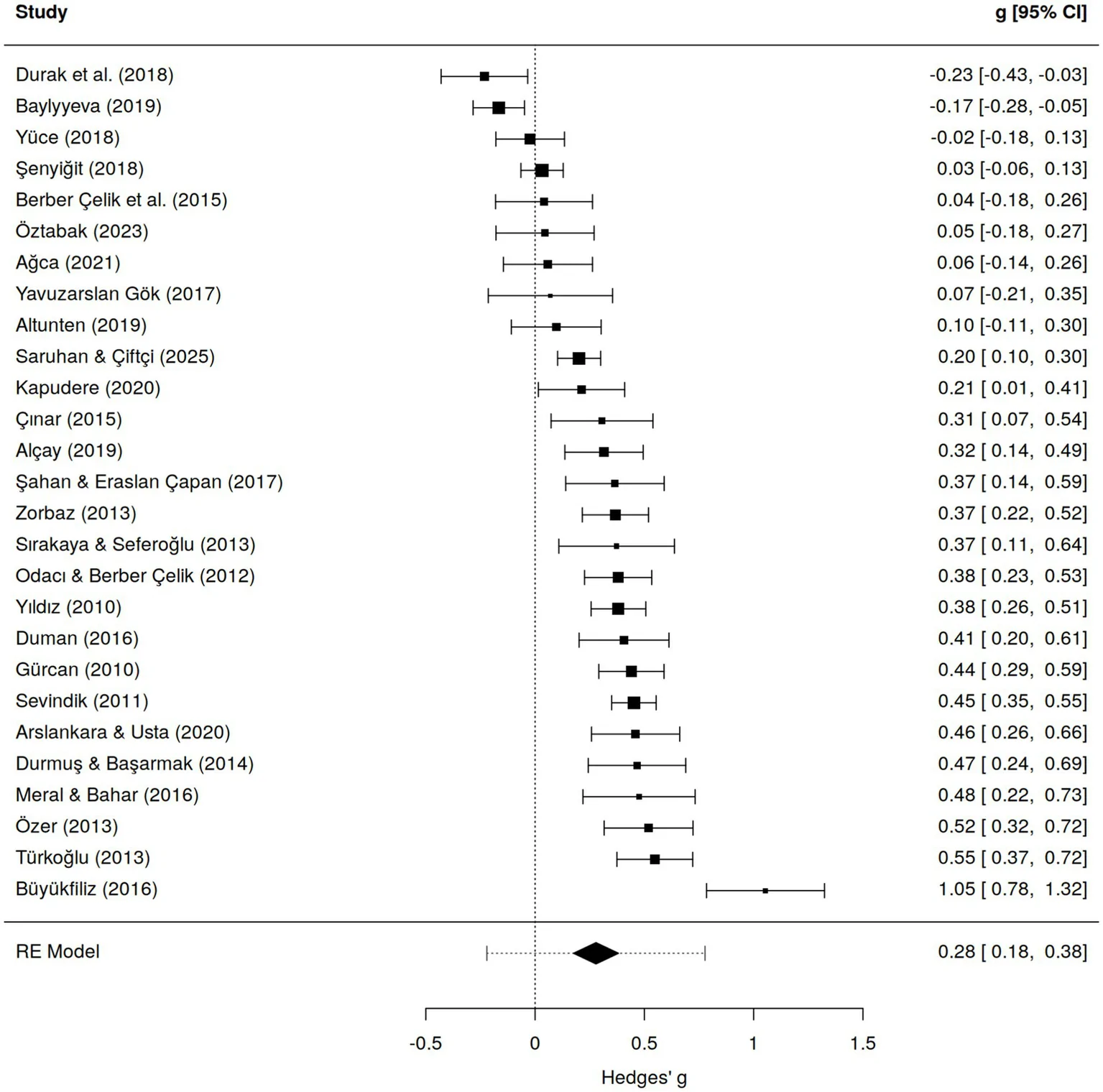
Forest plot of the gender comparison (male vs. female). The dotted line around the summary diamond shows the 95% prediction interval.

**Table 2:** Pooled effect sizes, heterogeneity and small-study effects.

Comparison	*k*	*N*	*g* [95% CI]	*p*	95% PI	*Q* (*df*)	*I*^2^ (%)	*τ* ^2^	Egger *z* (*p*)	Trim-and-fill: imputed; adjusted *g* [95% CI]
Gender (male vs. female)	27	16,479	0.28 [0.18, 0.38]	<0.001	[−0.22, 0.78]	215.07 (26)	88.8	0.056	1.20 (0.243)	0; 0.28 [0.18, 0.38]
Daily internet use (longest vs. shortest)	10	2,214	1.31 [1.03, 1.60]	<0.001	[0.43, 2.20]	58.76 (9)	82.4	0.136	2.24 (0.056)	1 (left); 1.25 [0.99, 1.51]
Family income (highest vs. lowest)	8	1,661	0.16 [−0.08, 0.39]	0.163	[−0.40, 0.71]	18.43 (7)	61.3	0.045	−2.37 (0.056)	3 (right); 0.29 [0.08, 0.51]

Random-effects models (REML) with Knapp–Hartung adjustment. N = number of participants in the two compared groups. PI = prediction interval. Egger’s test was implemented as a meta-regression on the standard error. Trim-and-fill was based on the REML model without the Knapp–Hartung adjustment. *p* < 0.05. *p* < 0.001.

Publication year moderated the effect (*b* = −0.031, *SE* = 0.012, *F*(1, 25) = 7.29, *p* = 0.012, *R*^2^ = 24.9%): the gender difference was smaller in more recent studies (Figure 3). Educational level was not a significant moderator (*F*(1, 25) = 2.79, *p* = 0.107). Leave-one-out estimates ranged from 0.25 to 0.30, and all confidence intervals excluded zero. Egger’s test was not significant (*z* = 1.20, *p* = 0.243), and trim-and-fill imputed no studies (Figure 4).

**Figure 3:**
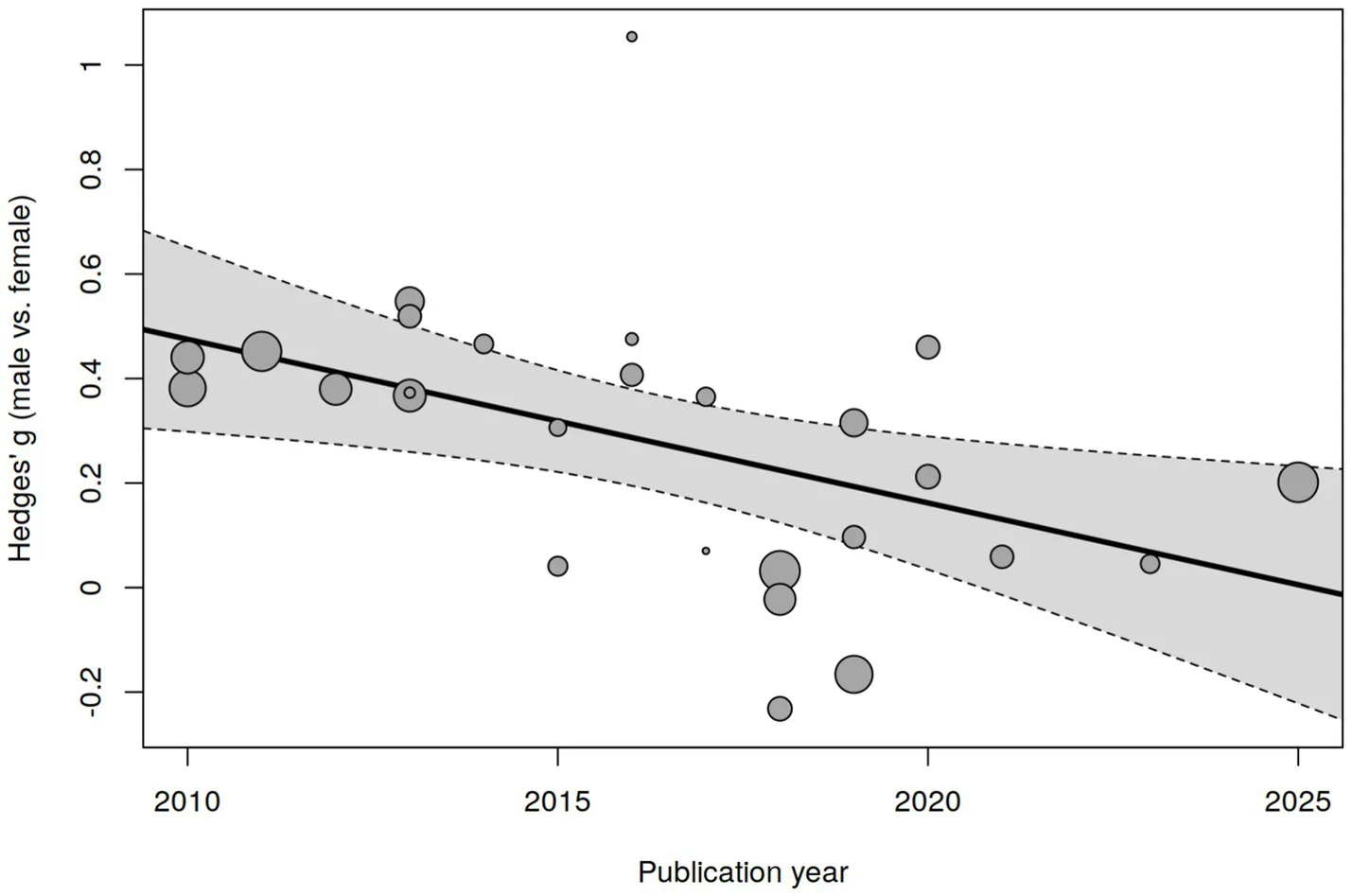
Meta-regression of the gender effect on publication year. Bubble size is proportional to study weight; the shaded band shows the 95% confidence interval of the regression line.

**Figure 4:**
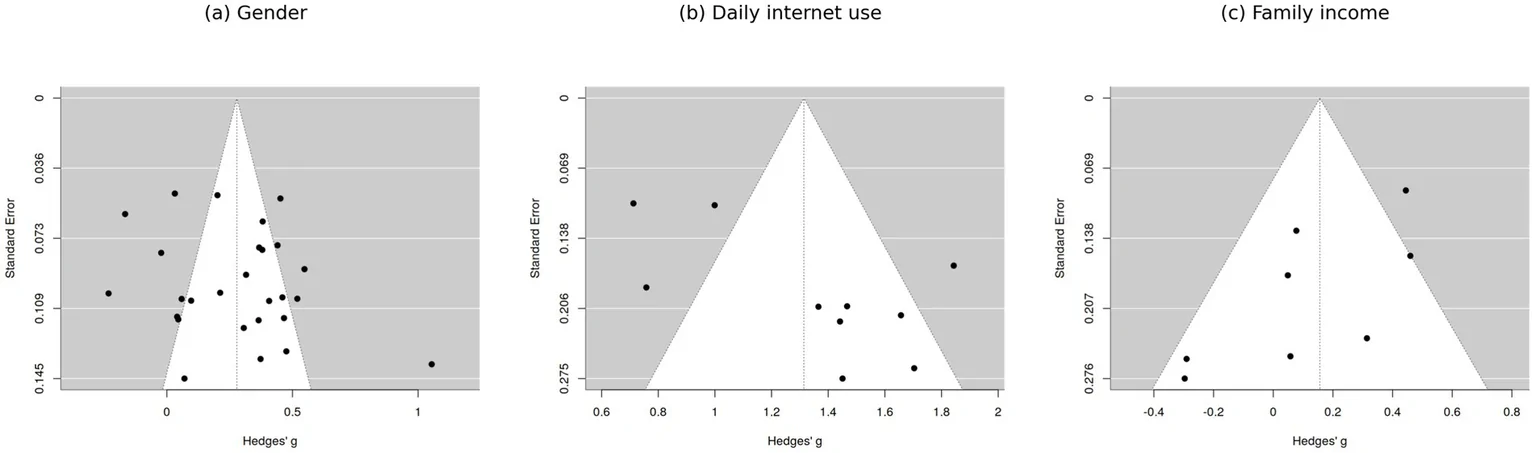
Funnel plots for **(A)** gender, **(B)** daily internet use, and **(C)** family income.

### 3.4 Daily internet use

Students in the longest daily-use category reported substantially higher PIU than those in the shortest category (*k* = 10, *N* = 2,214; *g* = 1.31, 95% CI [1.03, 1.60], *p* < 0.001; Figure 5). Heterogeneity was high (*Q*(9) = 58.76, *p* < 0.001; *I*^2^ = 82.4%; *τ*^2^ = 0.136), but the entire prediction interval was positive (0.43 to 2.20). Because the extreme categories differed across studies (e.g., “less than 1 hour” vs. “5 hours or more”; “3 hours or less” vs. “more than 3 hours”), the pooled estimate reflects contrasts between the lightest and heaviest users rather than a fixed dose difference.

**Figure 5:**
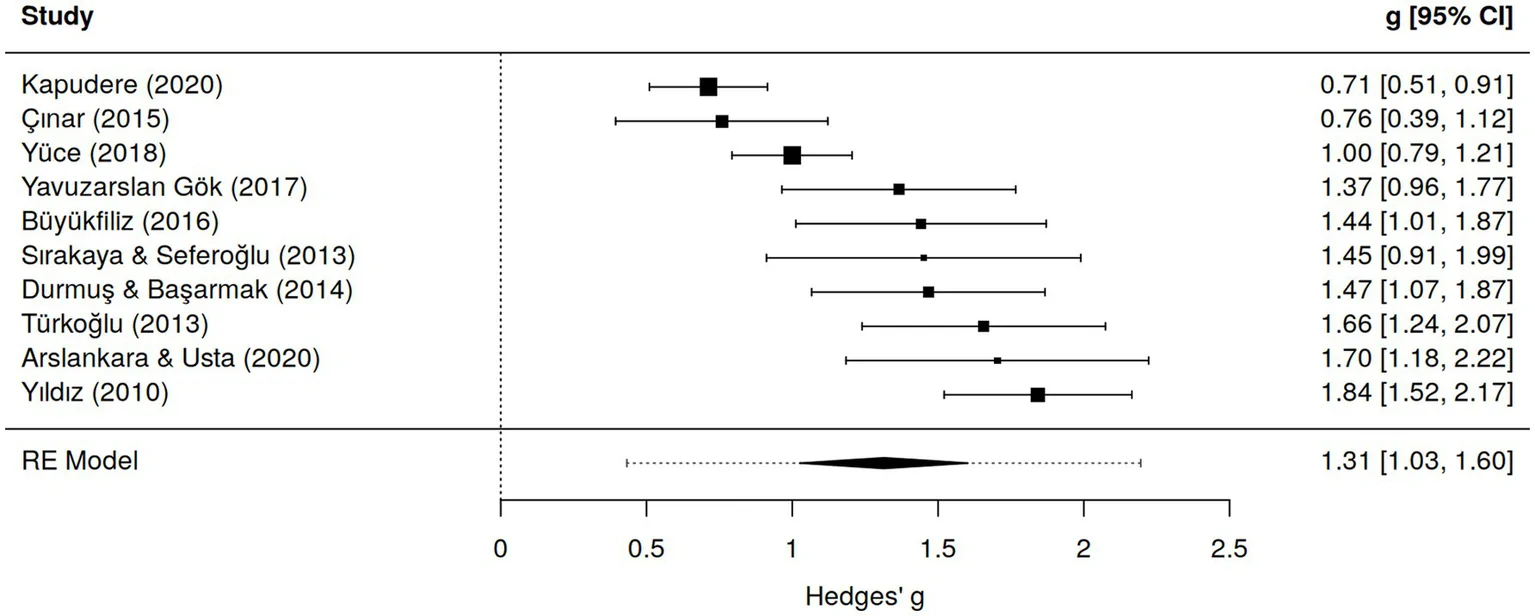
Forest plot of the daily internet use comparison (longest vs. shortest reported category).

Neither publication year (*F*(1, 8) = 4.29, *p* = 0.072) nor educational level (*F*(1, 8) = 0.26, *p* = 0.626) moderated the effect. Leave-one-out estimates ranged from 1.25 to 1.39, and excluding the two studies that reported weekly hours left the estimate unchanged (*k* = 8; *g* = 1.30, 95% CI [0.92, 1.68]). Egger’s test approached significance (*z* = 2.24, *p* = 0.056); trim-and-fill imputed one study on the left side of the funnel, and the adjusted estimate remained large (*g* = 1.25, 95% CI [0.99, 1.51]).

### 3.5 Family income

The difference in PIU between students from the highest and the lowest family-income categories was small and not statistically significant (*k* = 8, *N* = 1,661; *g* = 0.16, 95% CI [−0.08, 0.39], *p* = 0.163; Figure 6). Heterogeneity was moderate (*Q*(7) = 18.43, *p* = 0.010; *I*^2^ = 61.3%; *τ*^2^ = 0.045), and the prediction interval spanned zero (−0.40 to 0.71). No leave-one-out estimate reached significance (range 0.10 to 0.21), and publication year was not a moderator (*F*(1, 6) = 0.07, *p* = 0.806). The two university samples showed larger effects than the six high school samples (*F*(1, 6) = 18.27, *p* = 0.005), but because only two studies were in one subgroup, this contrast was not interpreted.

**Figure 6:**
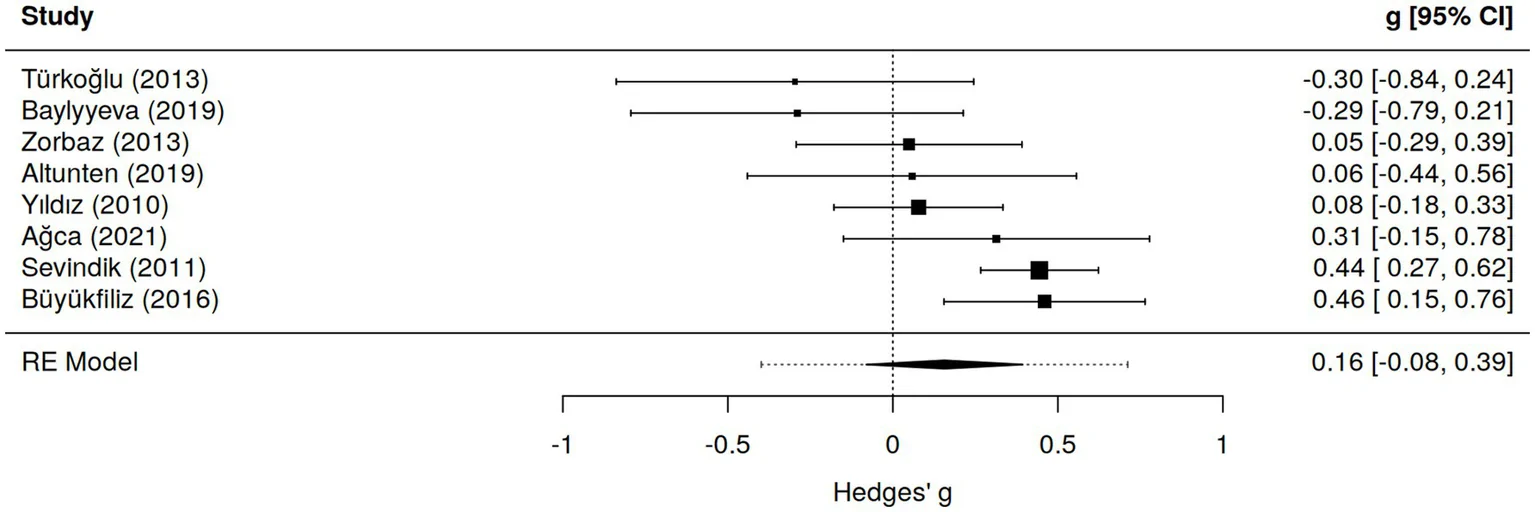
Forest plot of the family income comparison (highest vs. lowest reported category).

Egger’s test approached significance (*z* = −2.37, *p* = 0.056). Trim-and-fill imputed three studies on the right side of the funnel and yielded a larger adjusted estimate (*g* = 0.29, 95% CI [0.08, 0.51]). Given the small number of studies, interpret this adjustment with caution, and the evidence on family income remains inconclusive.

### 3.6 Sensitivity analyses

The pooled estimates were robust to the sensitivity analyses (Table 3). Restricting the gender analysis to the 25 fully verified studies (*g* = 0.29, 95% CI [0.19, 0.39]) or to the 20 originally coded studies (*g* = 0.28, 95% CI [0.19, 0.38]) did not change the conclusions, and the 12 studies reporting a sample-specific reliability coefficient yielded a similar estimate (*g* = 0.24, 95% CI [0.13, 0.36]). The corresponding subsets for daily use and family income contained only three studies each and are reported for completeness only.

**Table 3:** Moderator and sensitivity analyses.

Analysis	Gender	Daily internet use	Family income
Publication year: *b* (*SE*); *F*, *p*	−0.031 (0.012); *F*(1, 25) = 7.29, *p* = 0.012; *R*^2^ = 24.9%	−0.073 (0.035); *F*(1, 8) = 4.29, *p* = 0.072	−0.007 (0.029); *F*(1, 6) = 0.07, *p* = 0.806
University vs. high school: *b* (*SE*); *F*, *p*	0.19 (0.11); *F*(1, 25) = 2.79, *p* = 0.107	−0.14 (0.27); *F*(1, 8) = 0.26, *p* = 0.626	0.42 (0.10); *F*(1, 6) = 18.27, *p* = .005^a^
Leave-one-out, range of *g*	0.25–0.30	1.25–1.39	0.10–0.21
Fully verified studies only: *g* [95% CI] (*k*)	0.29 [0.19, 0.39] (25)	1.31 [1.03, 1.60] (10)	0.16 [−0.08, 0.39] (8)
Originally coded studies only: *g* [95% CI] (*k*)	0.28 [0.19, 0.38] (20)	—	—
Sample-specific α reported: *g* [95% CI] (*k*)	0.24 [0.13, 0.36] (12)	1.17 [−0.28, 2.62] (3)^b^	0.07 [0.03, 0.11] (3)^b^
Daily-hour categories only: *g* [95% CI] (*k*)	—	1.30 [0.92, 1.68] (8)	—

Mixed-effects meta-regressions (REML, Knapp–Hartung).

a Based on two university studies; not interpreted.

b Three studies only; reported for completeness.

## 4 Discussion

This meta-analysis combined 27 Turkish studies on differences in PIU among young people based on gender, daily internet usage, and family income. Time spent online showed the strongest link to PIU; the gender gap was modest and smaller in recent research; and income-related differences were minimal, non-significant, and influenced by small-study correction. These results are discussed using the cognitive-behavioral model of problematic internet use (Davis, 2001) and the I-PACE model (Brand et al., 2016; Brand et al., 2019).

### 4.1 Gender

Male students reported higher PIU than female students, consistent with earlier gender-focused meta-analyses (Akbaş et al., 2019; Öksüz, 2019). However, the effect was small, and the prediction interval included negative values; in two studies, female students reported higher PIU than male students (Baylyyeva, 2019; Durak et al., 2018). No evidence of small-study effects for gender.

Gender differences were less pronounced in more recent research. According to the I-PACE model, gender should be seen as a distal personal trait influencing PIU indirectly through usage motives and preferred online activities, rather than directly. Durar et al. (2024) found no gender difference in PIU among university students, although men mainly used the internet for videos and women for social media and communication. As smartphone-based social media use has become nearly universal across genders, disparities in exposure to motivating online activities may have decreased. Since the year effect reflects a between-study trend, it indicates a pattern across samples rather than individual changes and should be verified through repeated cross-sectional or longitudinal studies.

### 4.2 Daily internet use

Students in the highest daily internet usage group showed significantly higher PIU scores compared to those in the lowest group, with the entire prediction interval remaining positive. This aligns with Turkish research indicating that PIU tends to increase with daily internet consumption (Aras and Karakuş, 2025; Duman and Erdoğan Bakar, 2024; Durar et al., 2024), as well as multicenter studies showing that adolescents who spend over 7 h daily on social media face a higher risk of PIU (Yildirim Demirdöğen et al., 2024). According to cognitive-behavioral and I-PACE models, habitual use serves as the behavioral pathway through which users experience gratification and relief, thereby reinforcing cue-reactivity and habitual behaviors (Brand et al., 2019; Davis, 2001).

Three factors temper this interpretation. First, the PIUS includes an excessive-use dimension, so part of the association may reflect conceptual overlap between time spent online and the outcome measure. Secondly, using only the extreme categories from each study maximizes contrast, so the magnitude of g should not be interpreted as representing a fixed number of hours. Third, since the association is cross-sectional, it could be bidirectional: young people with PIU might spend more time online, or increased online time might contribute to PIU. Small-study analyses indicated limited asymmetry, but the adjusted estimate remained substantial.

### 4.3 Family income

PIU did not differ significantly between students from the highest- and lowest-income families. This aligns with existing research showing mixed effects of socioeconomic status on PIU. For example, higher household income and private internet access have been linked to increased PIU risk among Turkish high school students, supporting an access pathway (Sayılı et al., 2021). Conversely, family economic and educational factors are also considered risk factors through influences on family relationships and stress levels (Kabasakal and Akada, 2018). If both pathways are active, they may partially counterbalance each other overall; within the I-PACE model, income would serve as a distal contextual factor whose impact depends on its influence on access, parental mediation, and coping mechanisms.

Measurement limitations also restrict this analysis. Students provided income ranges in nominal Turkish lira, which varied significantly from 2010 to 2025. Additionally, the lower-income groups were often small. With only eight studies, the trim-and-fill adjustment-indicating a slight positive link—lacks reliability. Consequently, the evidence regarding family income should be seen as inconclusive rather than indicating no association.

### 4.4 Developmental stage and educational level

The 15–24 age group covers late adolescence and early emerging adulthood (Arnett, 2000). The analysis showed that educational level did not influence gender or daily-use effects, justifying the combination of high school and university samples for these comparisons. However, with only seven university samples and just two income-related samples, developmental differences might still exist. Future meta-analyses with more studies should explore smaller, more specific age ranges.

### 4.5 Implications

The strong, consistent link with daily internet use suggests that prevention efforts in schools and universities should target usage patterns such as time management, self-monitoring, and offline options rather than focusing on demographic risk groups. Given the small and possibly diminishing gender gap, gender-specific programs seem less necessary than those that address online behaviors and motivations tied to heavy usage. On a policy level, multi-stakeholder initiatives that integrate prevention, research, and youth input provide a useful model (Burkauskas, 2024). Current reviews emphasize the need for balanced, evidence-based approaches instead of reactions driven solely by concern (Odgers and Jensen, 2020).

### 4.6 Limitations

Several limitations should be acknowledged. First, all included studies were cross-sectional, so the results show associations rather than causality. Secondly, only studies using the PIUS or PIUS-A were considered. This enhanced comparability but excluded other instruments used to measure PIU, which vary in length and scoring. Thirdly, the categories for daily use and income differed among studies. Applying a single rule to compare extreme categories was not perfect, as some studies reported weekly hours instead of daily, and income brackets were given in nominal Turkish lira over a 15-year span.

Fourth, the analyses of daily use and income included only 10 and 8 studies, respectively, which limits moderator analyses and reduces power to detect small-study effects. Heterogeneity was high regarding gender and daily use, and was only partially explained by publication year. Fifth, 17 out of the 27 studies were graduate theses. While including theses helps reduce publication bias, it also increases variability in reporting quality: 15 studies reported reliability coefficients only from validation studies; 11 were excluded for lack of reliability data; and nine samples were from İstanbul. Sixth, the review protocol was not registered, no formal risk-of-bias tool was used, and for two studies, the extracted values could not be validated against the full texts. Sensitivity analyses indicated that excluding these studies did not alter the findings. Lastly, age reporting was inconsistent, with one high school sample including students aged 13 and 14.

## 5 Conclusion

In Türkiye, among young people, PIU is primarily linked to daily internet use. Male students tend to report slightly higher PIU than females, though this gap is small and seems to be decreasing. The role of family income remains unclear. Prevention strategies might be more effective if they focus on typical internet use behaviors rather than specific demographic groups.

Future research should (a) employ longitudinal studies to better understand the causal relationship between internet use over time and problematic internet use (PIU), (b) report internet usage as a continuous variable or through standardized categories, (c) assess socioeconomic status using consistent indicators like parental education or deprivation indices instead of just income brackets, (d) explore the mechanisms suggested by the cognitive-behavioral and I-PACE models, such as usage motives, coping strategies, and maladaptive thoughts, and (e) gather samples from regions outside major metropolitan areas.

## Data Availability

The original contributions presented in the study are included in the article/Supplementary material, further inquiries can be directed to the corresponding author.
