# Supplementary material for: Problematic internet use among young people in Türkiye: a meta-analysis
Source: Front Psychol. 2026 Oct 30;17:1885952. doi: 10.3389/fpsyg.2026.1885952 (PMC13759484; doi:10.3389/fpsyg.2026.1885952)
Supplement: Supplementary file 1 [file Supplementary_file_2.DOCX]

# Supplementary File S2 — PRISMA 2020 Checklist (Ms. 1885952)

Locations refer to section numbers of the revised manuscript; page or line numbers should be added once the final file is paginated. Item wording is abbreviated; the official PRISMA 2020 template (Page et al., 2021) can be used for submission with the locations below.

| Section | Item | Checklist item (abbreviated) | Location in revised manuscript |
| --- | --- | --- | --- |
| Title | 1 | Identify the report as a systematic review | Title (“…: A meta-analysis”); article type: Systematic Review |
| Abstract | 2 | Structured abstract following PRISMA 2020 for Abstracts | Abstract |
| Introduction | 3 | Rationale in the context of existing knowledge | Introduction, paragraphs 1–6 |
| Introduction | 4 | Objectives or questions addressed | Introduction, final paragraph (three research questions and moderator aims) |
| Methods | 5 | Eligibility criteria and how studies were grouped for syntheses | Section 2.2 (PECOS); Section 2.5 |
| Methods | 6 | Information sources and date last searched | Section 2.3 |
| Methods | 7 | Full search strategies | Section 2.3 |
| Methods | 8 | Selection process (screeners, independence, automation tools) | Sections 2.3–2.4 |
| Methods | 9 | Data collection process | Section 2.4 |
| Methods | 10a | Outcomes for which data were sought | Sections 2.2 and 2.4 |
| Methods | 10b | Other variables for which data were sought | Section 2.4; Supplementary Table S1 |
| Methods | 11 | Methods to assess risk of bias in included studies | Section 2.6 (quality indicators; no formal risk-of-bias tool) |
| Methods | 12 | Effect measures used | Section 2.5 (Hedges’ *g*) |
| Methods | 13a | How studies were selected for each synthesis | Sections 2.2 and 2.5 |
| Methods | 13b | Data preparation (e.g., handling missing statistics, conversions) | Section 2.5 (extreme-category rule; weekly hours) |
| Methods | 13c | Methods to tabulate or display results | Section 2.7; Tables 1–3; Figures 2–6 |
| Methods | 13d | Synthesis methods, model, heterogeneity, software | Section 2.7 |
| Methods | 13e | Methods to explore heterogeneity | Section 2.7 (meta-regression on year and educational level) |
| Methods | 13f | Sensitivity analyses | Section 2.7 |
| Methods | 14 | Methods to assess risk of reporting bias | Section 2.7 (funnel plots, Egger’s test, trim-and-fill) |
| Methods | 15 | Methods to assess certainty of evidence | Not performed |
| Results | 16a | Results of the search and selection process (flow diagram) | Section 3.1; Figure 1 |
| Results | 16b | Studies that appeared eligible but were excluded, with reasons | Section 3.1 |
| Results | 17 | Characteristics of included studies | Section 3.2; Table 1; Supplementary Table S1 |
| Results | 18 | Risk of bias in included studies | Section 3.2; Supplementary Table S1 (quality indicators) |
| Results | 19 | Results of individual studies | Table 1; Figures 2–4 |
| Results | 20a | Characteristics and risk of bias of contributing studies per synthesis | Sections 3.2–3.5; Table 1 |
| Results | 20b | Results of all statistical syntheses | Sections 3.3–3.5; Table 2 |
| Results | 20c | Investigations of possible causes of heterogeneity | Sections 3.3–3.5; Table 3; Figure 5 |
| Results | 20d | Sensitivity analyses | Section 3.6; Table 3 |
| Results | 21 | Risk of bias due to missing results (reporting biases) | Sections 3.3–3.5; Table 2; Figure 6 |
| Results | 22 | Certainty of evidence | Not assessed |
| Discussion | 23a | General interpretation in the context of other evidence | Sections 4.1–4.4 |
| Discussion | 23b | Limitations of the evidence | Section 4.6 |
| Discussion | 23c | Limitations of the review processes | Section 4.6 |
| Discussion | 23d | Implications for practice, policy and future research | Section 4.5; Section 5 |
| Other information | 24a | Registration information | Section 2.1 (protocol not registered) |
| Other information | 24b | Where the protocol can be accessed | Not applicable (no protocol prepared in advance) |
| Other information | 24c | Amendments to registration or protocol | Not applicable |
| Other information | 25 | Sources of financial or non-financial support | Funding statement |
| Other information | 26 | Competing interests | Conflict of interest statement |
| Other information | 27 | Availability of data, code and other materials | Section 2.7; Data availability statement; Supplementary Files S3–S4 |

*Reference.* Page, M. J., McKenzie, J. E., Bossuyt, P. M., Boutron, I., Hoffmann, T. C., Mulrow, C. D., Shamseer, L., Tetzlaff, J. M., Akl, E. A., Brennan, S. E., Chou, R., Glanville, J., Grimshaw, J. M., Hróbjartsson, A., Lalu, M. M., Li, T., Loder, E. W., Mayo-Wilson, E., McDonald, S., … Moher, D. (2021). The PRISMA 2020 statement: An updated guideline for reporting systematic reviews. *BMJ, 372*, Article n71. https://doi.org/10.1136/bmj.n71
